# Contrasting requirements during disease evolution identify EZH2 as a therapeutic target in AML

**DOI:** 10.1084/jem.20181276

**Published:** 2019-03-19

**Authors:** Faisal Basheer, George Giotopoulos, Eshwar Meduri, Haiyang Yun, Milena Mazan, Daniel Sasca, Paolo Gallipoli, Ludovica Marando, Malgorzata Gozdecka, Ryan Asby, Olivia Sheppard, Monika Dudek, Lars Bullinger, Hartmut Döhner, Richard Dillon, Sylvie Freeman, Oliver Ottmann, Alan Burnett, Nigel Russell, Elli Papaemmanuil, Robert Hills, Peter Campbell, George S. Vassiliou, Brian J.P. Huntly

**Affiliations:** 1 Wellcome Trust–Medical Research Council Cambridge Stem Cell Institute, Cambridge, UK; 2 Department of Haematology, University of Cambridge, Cambridge, UK; 3 Cambridge Institute for Medical Research, Cambridge Biomedical Campus, Cambridge, UK; 4 Wellcome Trust Sanger Institute, Hinxton, UK; 5 Charité University Hospital, Berlin, Germany; 6 Department of Internal Medicine III, University of Ulm, Ulm, Germany; 7 Department of Medical and Molecular Genetics, Kings College School of Medicine, UK; 8 Department of Clinical Immunology, University of Birmingham Medical School, Edgbaston, Birmingham, UK; 9 Department of Haematology, University of Cardiff, Cardiff, UK; 10 Isle of Arran, UK; 11 Department of Haematology, University of Nottingham, Nottingham, UK; 12 Departments of Epidemiology and Biostatistics and Cancer Biology, the Center for Molecular Oncology and the Center for Hematologic Malignancies, Memorial Sloan-Kettering Cancer Center, New York, NY; 13 Nuffield Department of Population Health, University of Oxford, Oxford, UK

## Abstract

Epigenetic regulators, such as EZH2, are frequently mutated in cancer, and loss-of-function *EZH2* mutations are common in myeloid malignancies. We have examined the importance of cellular context for Ezh2 loss during the evolution of acute myeloid leukemia (AML), where we observed stage-specific and diametrically opposite functions for Ezh2 at the early and late stages of disease. During disease maintenance, WT Ezh2 exerts an oncogenic function that may be therapeutically targeted. In contrast, Ezh2 acts as a tumor suppressor during AML induction. Transcriptional analysis explains this apparent paradox, demonstrating that loss of *Ezh2* derepresses different expression programs during disease induction and maintenance. During disease induction, *Ezh2* loss derepresses a subset of bivalent promoters that resolve toward gene activation, inducing a feto-oncogenic program that includes genes such as *Plag1*, whose overexpression phenocopies *Ezh2* loss to accelerate AML induction in mouse models. Our data highlight the importance of cellular context and disease phase for the function of Ezh2 and its potential therapeutic implications.

## Abstract (precis)

Basheer et al. show that EZH2 has stage-specific and diametrically opposite roles during the induction and maintenance stages of AML. However, different transcriptional programs are affected at each stage, identifying mutant *EZH2* as a prognostic marker and paradoxically WT EZH2 as a potential therapeutic target.

## Introduction

Acute myeloid leukemia (AML) is an aggressive hematological cancer with a dismal outlook (Ferrara and Schiffer, 2013). Recently, characterization of the mutational landscape of AML has allowed for a deeper understanding of its biology (Grimwade et al., 2016; Papaemmanuil et al., 2016) and the identification of potentially sensitive genotypes, facilitating the development of novel agents that target them and providing promise of improved outcomes (Daigle et al., 2011; Dawson et al., 2011, 2012; Basheer and Huntly, 2015; Gallipoli et al., 2015, 2018; Giotopoulos et al., 2016). Enhancer of zeste homologue 2 (EZH2) forms the core of the multiprotein Polycomb repressive complex 2 (PRC2; Margueron and Reinberg, 2011). It is responsible for the transcriptional repression of target genes by depositing di- and trimethylation on lysine 27 of histone 3 (H3K27me3) through its catalytic SET domain at proximal and distal regulatory elements (Cao et al., 2002; Kuzmichev et al., 2002), with the H3K27me3 mark repressing gene expression through a number of mechanisms (Wang et al., 2004; Barski et al., 2007; Hansen et al., 2008; Simon and Kingston, 2013). Aberrant EZH2 expression and activity have also been linked to tumorigenesis; *EZH2* was found to be overexpressed in breast, prostate, and renal cancers, where its levels correlate with poor prognosis (Varambally et al., 2002; Kleer et al., 2003; Wagener et al., 2008). Gain-of-function mutations of *EZH2* at codon Y641 have also been described in between 7% and 22% of patients with follicular lymphoma and diffuse large B cell lymphomas, respectively (Morin et al., 2010), and promising preclinical efficacy with small-molecule inhibitors of EZH2 methyltransferase activity has led to ongoing phase 1 trials (NCT02082977, NCT01897571, and NCT02395601), the results of which are awaited. In contrast, the role of EZH2 in myeloid malignancies is less well defined and at first glance counterintuitive. Predominantly hemizygous, predicted loss-of-function mutations have been described at low frequency in myeloid malignancies, including myeloproliferative neoplasms (MPNs), myelodysplasia (MDS), and AML (Ernst et al., 2010; Makishima et al., 2010; Nikoloski et al., 2010; Ley et al., 2013), and *Ezh2* loss in mouse models has been shown to lead to the development of multiple long-latency hematological malignancies, predominantly MDS, MPN, and T-adult lymphoblastic leukemia/T cell lymphoma (Simon et al., 2012; Mochizuki-Kashio et al., 2015). Moreover, loss of *Ezh2* accelerates the development of myelofibrosis and decreases survival in *Jak2*-V617F–driven MPN (Sashida et al., 2016; Shimizu et al., 2016; Yang et al., 2016) and *Runx1* mutated MDS (Sashida et al., 2014), identifying *EZH2* as a tumor suppressor. However, contrary to this role, reports have also demonstrated that EZH2 is required for chronic myeloid leukemia stem cell function (Scott et al., 2016; Xie et al., 2016), and two separate studies have suggested that maintenance of MLL-AF9 AML is reliant on *Ezh2* (Neff et al., 2012; Tanaka et al., 2012), suggesting EZH2 carries oncogenic function and is therefore a plausible therapeutic target in this context. Given the counterintuitive data on the function of EZH2 within myeloid malignancies, we sought to explore this further in the context of AML and delineate the role of EZH2 across different AML subtypes, as well as during different phases of the disease. In this study, using genetic and pharmacological models, we demonstrate that Ezh2 clearly has contrasting roles at different disease stages—a tumor-suppressive function during leukemogenesis and an oncogenic function during leukemia maintenance—the first such demonstration for an epigenetic regulator. Moreover, we provide mechanisms for its tumor-suppressor role in our AML models and demonstrate a minimal overlap between genes that mediate the tumor-suppressive and oncogenic functions. Taken together, these data provide reassurance that despite its tumor-suppressive effects during leukemogenesis, EZH2 may be a promising therapeutic target in established AML.

## Results

### Ezh2 is required for the maintenance of multiple AML genotypes

**Figure 1.**
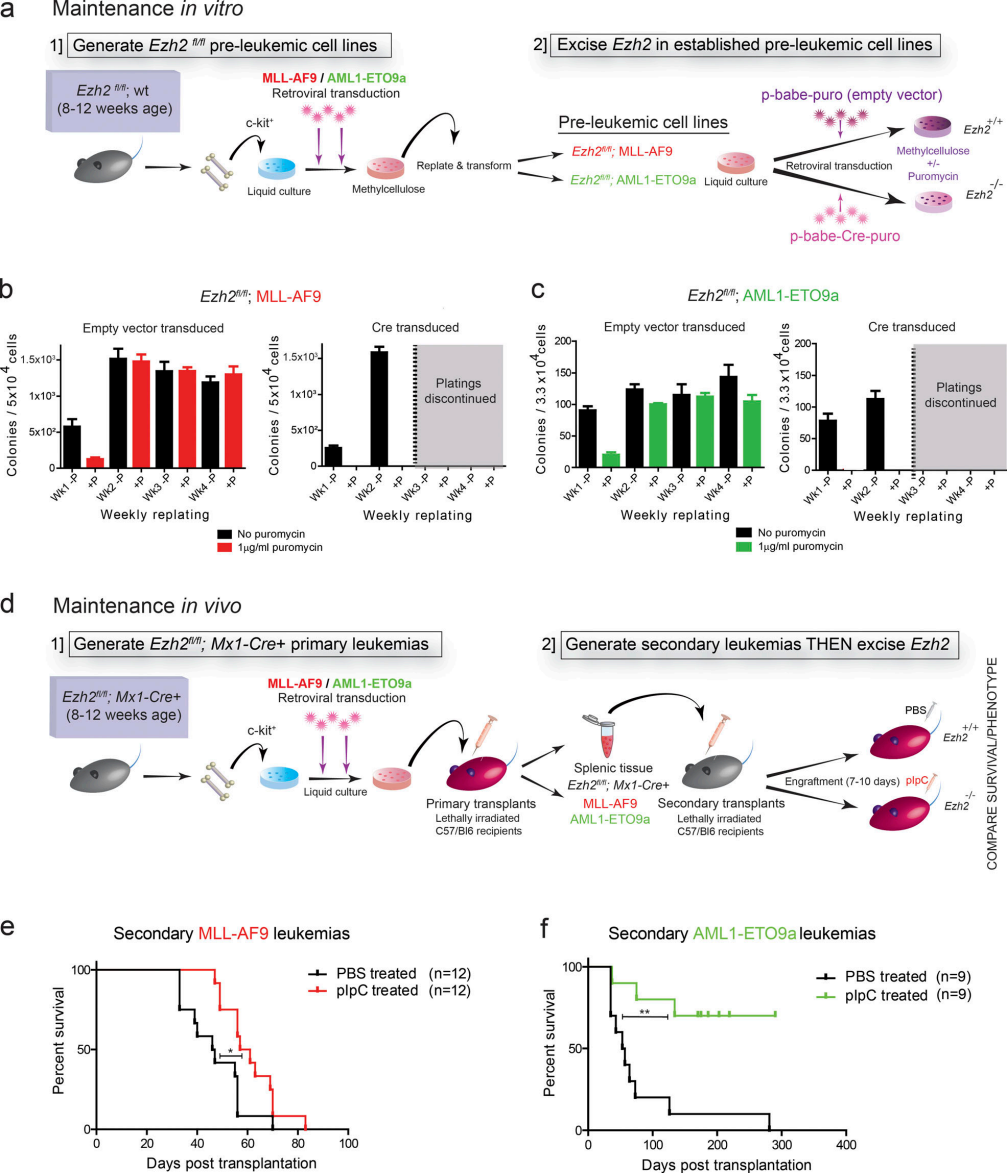
***Ezh2* functions as an oncogene during the maintenance of disparate subtypes of AML in vitro and in vivo*.* (a)** Schema of the in vitro experiments. (1) *Ezh2^fl/fl^* immortalized cell lines were generated from *Ezh2^fl/fl^*; WT mice at 8–12 wk of age via retroviral transduction with *MLL-AF9* or *AML1-ETO9a* individually and were allowed to immortalize over successive methylcellulose replatings. (2) *Ezh2* was excised in established preleukemic cell lines via a *p-Babe-Cre-puro* vector (for deletion of *Ezh2* in vitro) or a *p-Babe-puro* empty vector (control) and cultured ± puromycin in methylcellulose. **(b and c)** Methylcellulose replatings for empty vector–transduced vs. *p-babe-Cre-puro*–transduced *Ezh2^fl/fl^; MLL-AF9* immortalized murine c-kit^+^ BM HSPCs (b) and *Ezh2^fl/fl^*; *AML1-ETO9a* (c) immortalized cell lines in the absence or presence of puromycin to select positively transduced cells. *p-babe-Cre-puro*–transduced cells fail to form colonies and exhaust at the first round of replating (while empty vector–transduced cells expand and form colonies on iterative replatings), indicating an absolute requirement of *Ezh2* for maintenance of *MLL-AF9* and *AML1-ETO9a* in vitro (*n* = 2 independent experiments). **(d)** Schematic diagram of the in vivo experimental process. (1) Generate *Ezh2^fl/fl^; Mx1-Cre*^+^ primary leukemias: c-kit^+^ BM HSPCs harvested from *Ezh2^fl/fl^*; *Mx1-Cre*^+^ were transduced with either *MLL-AF9* or *AML1-ETO9a* retrovirus and then transplanted into lethally irradiated, WT C57/Bl6 recipients. Following development of leukemia, BM and spleen were harvested and stored. (2) Secondary leukemias were generated, and following engraftment, *Ezh2* was excised in situ via intraperitoneal pIpC injections (vs. intraperitoneal PBS as control). **(e)** Kaplan–Meier graph demonstrating significantly increased survival for *Ezh2* excised *MLL-AF9* secondary leukemias (*n* = 12 animals) following administration of pIpC over PBS-treated *Ezh2^fl/fl^* MLL-AF9 secondary leukemias (*n* = 12 animals, log-rank [Mantel–Cox] test P = 0.0163). **(f)** Kaplan–Meier graph demonstrating significantly increased survival of *Ezh2* excised AML1-ETO9a secondary leukemias (*n* = 9 animals) following administration of pIpC- over PBS-treated *Ezh2^fl^*^/fl^ AML1-ETO9a secondary leukemias (*n* = 9 animals, log-rank [Mantel–Cox] test P = 0.0011). *, P < 0.05; **, P < 0.01. All error bars are ± SEM.

To dissect the effects of *Ezh2* loss during AML evolution, we targeted EZH2 at various experimental time points using both genetic ablation and pharmacological inhibition. We used retroviral overexpression of two AML fusion oncogenes (*MLL-AF9* and *AML1-ETO9a*) that generate separate and disparate models of AML, are associated with a clinical range from good to poor prognosis, and markedly differ in their mechanisms of leukemic transformation. Initially focusing on the role of *Ezh2* in AML maintenance, we generated cell lines immortalized by retroviral expression of *MLL-AF9* or *AML1-ETO9a* in hematopoietic stem and progenitor cells (HSPCs) from *Ezh2^fl/fl^* and WT mice. Through retroviral expression of Cre (*p-babe-Cre-puro* or the empty control vector *p-babe-puro*), we then deleted *Ezh2*, generating an *Ezh2*^−/−^ and *Ezh2*^+/+^ genotype cellular background for each oncogene, respectively (Fig. 1 a). Strikingly, we could demonstrate that continued expression of *Ezh2* was an absolute requirement for the continued in vitro propagation of both immortalized cell lines in serial methylcellulose replating experiments (Fig. 1, b and c; and Fig. S1), with colony formation completely abrogated in *Ezh2^−/−^* cells from the first plating onward. This was in stark contrast to empty vector–transduced (*Ezh2^+/+^*) cells, which iteratively replated. Furthermore, the effects of Cre-mediated toxicity and/or low transduction efficiency were excluded as Cre-transduced *Ezh2^wt/wt^; MLL-AF9* or *AML1-ETO9a*–transformed cells were also able to repopulate and form colonies adequately (Fig. S1 and data not shown).

To evaluate the role of *Ezh2* in AML maintenance in vivo, and therefore determine the therapeutic implications of targeting EZH2 in established AML, we then generated primary *MLL-AF9* and *AML1-ETO9a* murine leukemias on an *Ezh2^fl/fl^;Mx1-Cre* background but did not treat donor or recipient mice with poly(I)-poly(C) (pIpC), thus leaving the *Ezh2* locus intact (Fig. 1 d). Secondary leukemias were then generated using transplantation of primary splenic tissue into WT lethally irradiated recipients, and *Ezh2* deletion was achieved in situ in the test mice, following adequate engraftment, by the administration of pIpC (or PBS injections in the control arm) in recipient mice. We were able to reproduce previous data that deletion of *Ezh2* significantly disrupted the progression and prolonged survival of secondary *MLL-AF9* leukemias (Neff et al., 2012; Tanaka et al., 2012). Moreover, and in keeping with our in vitro findings, we extended this oncogenic role for Ezh2, further demonstrating similar effects in *AML1-ETO9a* leukemias in vivo (Fig. 1, e and f, respectively). There were no demonstrable differences in disease bulk or pattern of infiltration between *Ezh2^+/+^* and *Ezh2^−/−^* recipient mice (Fig. S2). Of note, in the *AML-ETO9a Ezh2^−/−^* cohort, only three of ten animals actually developed leukemia, with the other animals succumbing to either thymic lymphomas or other nonleukemic causes of death only after long latency (Table S1). Taken together, these data suggest that *Ezh2* displays oncogenic function across disparate subtypes of established AML and suggest it as a possible therapeutic target across AML subtypes.

### Validation of EZH2 as a therapeutic target in AML models and patient samples

**Figure 2.**
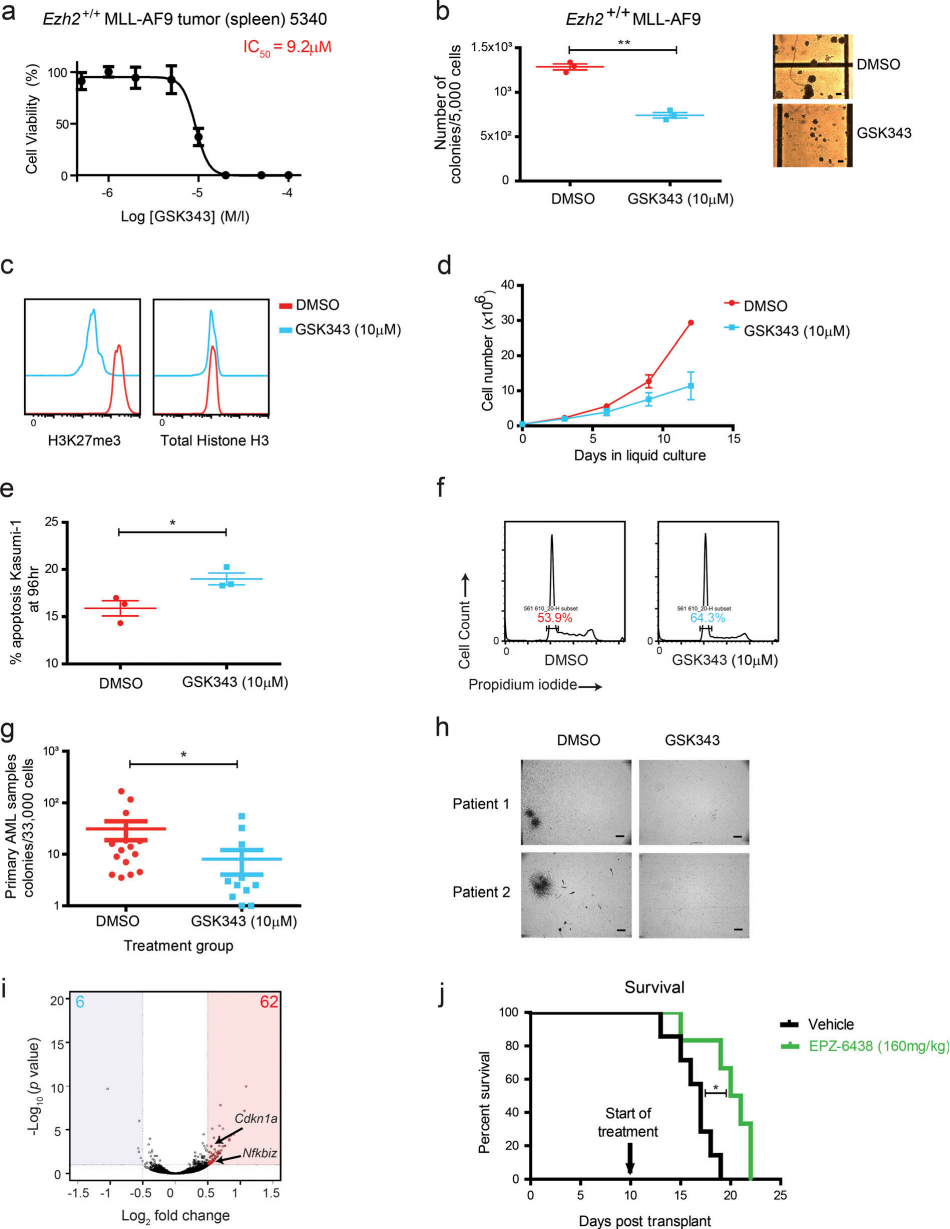
**Ezh2/EZH2 is a viable therapeutic target across multiple AML genotypes and in primary AML samples. (a)** Representative cell proliferation assay plot demonstrating *Ezh2^+/+^*; *MLL-AF9* tumor splenocytes cultured in liquid medium exhibit sensitivity to the EZH2 inhibitor GSK343 with an IC_50_ value of ∼10 µM. **(b)** Left: Clonogenic assay for *Ezh2^+/+^; MLL-AF9* tumor splenocytes performed in the presence of DMSO (vehicle control) or 10 µM GSK343 (performed in triplicate, P = 0.0003, two-tailed *t* test). Right: Photomicrographs demonstrating reduction in colony size upon treatment with GSK343 compared with DMSO (bars, 100 µm). **(c)** Flow cytometry at 24 h for H3K27me3 and total H3 demonstrates that 10 µM GSK343 significantly reduces H3K27me3 over DMSO treatment in *Ezh2^+/+^; MLL-AF9* tumor splenocytes cultured in liquid medium. **(d)** Liquid culture growth assay for human AM1-ETO–positive cell line Kasumi-1 over a 12-d time course in the presence of 10 µM GSK343 compared with DMSO (performed in duplicate). **(e)** 10 µM GSK343 treatment leads to a modest increase in apoptosis at late time points (96 h) in the Kasumi-1 cell line compared with DMSO (P = 0.037, two-tailed *t* test, performed in triplicate). **(f)** Representative cell cycle analysis plot at 96 h in Kasumi-1 also demonstrates mild G_0_/G_1_ cell cycle arrest following 10 µM GSK343 treatment. **(g)** Colony assays across a wide range of AML patient samples (*n* = 15) demonstrates a significant reduction in colony numbers following treatment with 10 µM GSK343 compared with DMSO. Cases include varying molecular subtypes, with variable karyotypic mutational and prognostic status. (P = 0.01, two-tailed *t* test). **(h)** Photomicrographs of two different human primary AML samples in a clonogenic assay cultured with either DMSO or 10 µM GSK343 (bars, 500 µm). **(i)** Volcano plot for DMSO- vs. 10 µM GSK343–treated *Ezh2^+/+^; MLL-AF9* tumor splenocytes cultured in liquid medium demonstrating gene expression changes and showing the adjusted significance P value (log_10_) vs. fold change (log_2_; *n* = 2, biological replicates for DMSO and GSK343). Potential candidates that might underlie the antileukemic effects, *Cdkn1a* and *Nfkbiz*, are identified. **(j)** Kaplan–Meier graph of survival of secondary transplants of AML1-ETO9a AML generated from *Ezh2^+/+^* mice comparing EPZ-6438–treated (*n* = 7) and vehicle control–treated (*n* = 6; log-rank [Mantel–Cox] test P = 0.0103). *, P < 0.05; **, P < 0.01. All error bars are ± SEM.

To test this hypothesis, we next sought to examine the effects of pharmacological inhibition of EZH2 in AML, using GSK343, an S-adenosyl methionine–competitive EZH2 inhibitor (Verma et al., 2012). Murine *Ezh2^+/+^ MLL-AF9* spleen tumor cells cultured in vitro demonstrated sensitivity to GSK343 with an IC_50_ (concentration where 50% maximal growth inhibition is observed) of ∼10 µM (Fig. 2 a) and showed a significant reduction in colony size and formation in its presence compared with vehicle control (Fig. 2 b). We correlated GSK343’s effects to direct inhibition of EZH2 enzymatic function, demonstrating a decrease in H3K27me3 in these tumor cells as early as 24 h after treatment (Fig. 2 c). Consistent with our genetic studies, the human AML1-ETO-fusion-driven cell line Kasumi-1 also demonstrated increased sensitivity to GSK343 at similar IC_50_ to the murine AML tumors (Fig. S3 a), with marked reduction in liquid culture expansion compared with vehicle control (Fig. 2 d). Functional analysis revealed that EZH2 inhibition with GSK343 resulted in a modest increase in apoptosis and G_0_/G_1_ cell cycle arrest at 96 h into treatment (Fig. 2, e and f). Transformed *Ezh2^+/+^ MLL-AF9* murine bone marrow (BM) HSPCs also had reduced clonogenic potential with decreased colony formation when treated with GSK343 (Fig. S3 b). We further tested EZH2 inhibition in primary leukemia cells isolated from patients across various AML genotypes (*n* = 15; Fig. 2, g and h; and Fig. S3 c), where significant reduction of colony formation was seen, in contrast to the minimal effects of GSK343 on the clonogenic function of normal primary CD34^+^ hematopoietic stem cells (HSCs; *n* = 3; Fig. S3 d). Gene expression differences in GSK343 vs. control-treated *MLL-AF9* tumors in vitro was assessed through RNA sequencing (RNA-seq; Fig. 2 i and Table S2), with a relatively narrow set of genes deregulated following Ezh2 inhibition with GSK343 (62 up-regulated or “derepressed” genes vs. 6 down-regulated). Among the genes derepressed following Ezh2 inhibition were the cyclin-dependent kinase inhibitor *Cdkn1a* (*p21Cip1*), which has previously been shown to be up-regulated following genetic loss of *Ezh2* (Tanaka et al., 2012), and other genes whose up-regulation may explain, at least in part, the antileukemic effects, including the NF-κB inhibitor *Nfkbiz.* To further assess the value of therapeutically targeting EZH2, particularly in AML1-ETO9a disease, where we saw the greatest effect in our genetic in vivo experiment, we developed AML1-ETO9a secondary mouse leukemias and undertook an in vivo drug trial of EZH2 inhibition, using the compound EPZ-6438, a clinically relevant EZH2 inhibitor currently in early-phase trials (NCT01897571). We adopted an oral dosing strategy as previously described (Knutson et al., 2014) and compared survival outcomes against mice treated with vehicle control. In consonance with our in vitro and in vivo genetic disruption experiments in AML1-ETO9a and preclinical pharmacological observations, treatment with EPZ-6438 prolonged the survival of AML1-ETO9a secondary leukemic mice compared with those treated with vehicle control (Fig. 2 j). These data corroborate our genetic studies and suggest therapeutic potential and a realistic therapeutic window, identifying EZH2 as a valid target across multiple AML genotypes.

### EZH2 is a tumor suppressor during AML induction, and its mutation confers a poor prognosis

**Figure 3.**
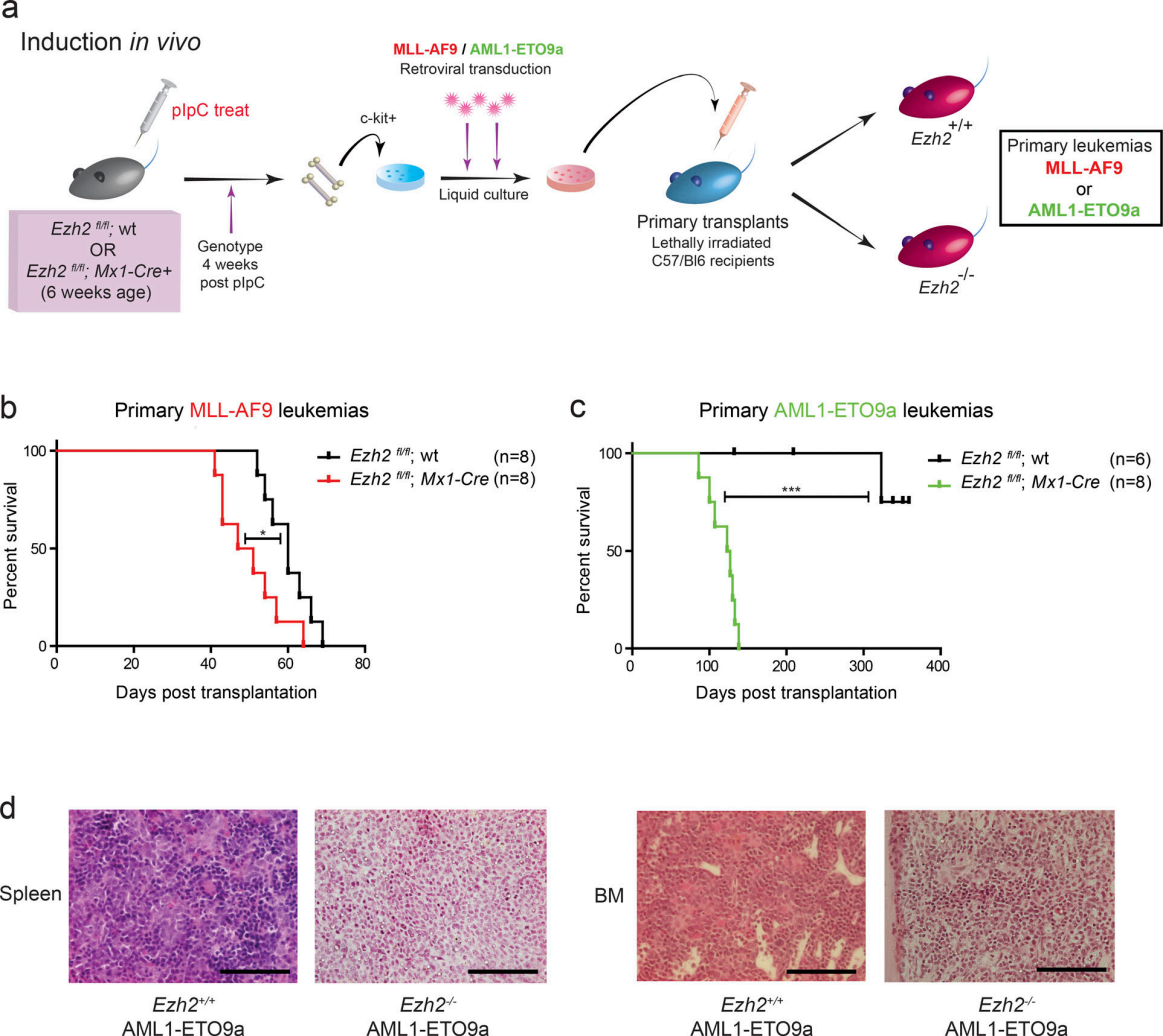
***Ezh2* functions as a tumor suppressor during the induction of disparate subtypes of AML in vivo*.* (a)** Schema of the in vivo experiments. *Ezh2^fl/fl^*; WT or *Ezh2^fl/fl^*; *Mx1-Cre^+^* mice were treated with pIpC to induce *Ezh2* deletion in *Mx1-Cre*–expressing mice before retroviral transduction with either *MLL-AF9* or *AML1-ETO9a* retrovirus followed by transplantation into lethally irradiated WT C57/Bl6 recipient mice. **(b)** Kaplan–Meier graph demonstrating significantly increased survival for *Ezh2^+/+^*; *MLL-AF9* (WT) primary leukemias (*n* = 8 animals) vs. *Ezh2^−/−^*; *MLL-AF9* primary leukemias (*n* = 8 animals, log-rank [Mantel–Cox] test P = 0.0341). **(c)** Kaplan–Meier graph demonstrating significantly increased survival for *Ezh2^+/+^*; *AML1-ETO9a* (WT) primary leukemias (*n* = 6 animals) vs. *Ezh2^−/−^*; *AML1-ETO9a* primary leukemias (*n* = 8 animals, log-rank [Mantel–Cox] test P = 0.0004). **(d)** Histopathology of spleen (left) and BM (right) taken at necropsy in *Ezh2^+/+^* vs. *Ezh2^−/−^ AML1-ETO9a* murine primary leukemias. Both samples show obvious and similar degrees of leukemic infiltration with large primitive blast cells that demonstrated a myeloid phenotype on flow cytometry, with no macroscopic or microscopic phenotypic difference demonstrated between the leukemias of either genotype (bars, 100 µm). *, P < 0.05; ***, P < 0.001.

**Figure 4.**
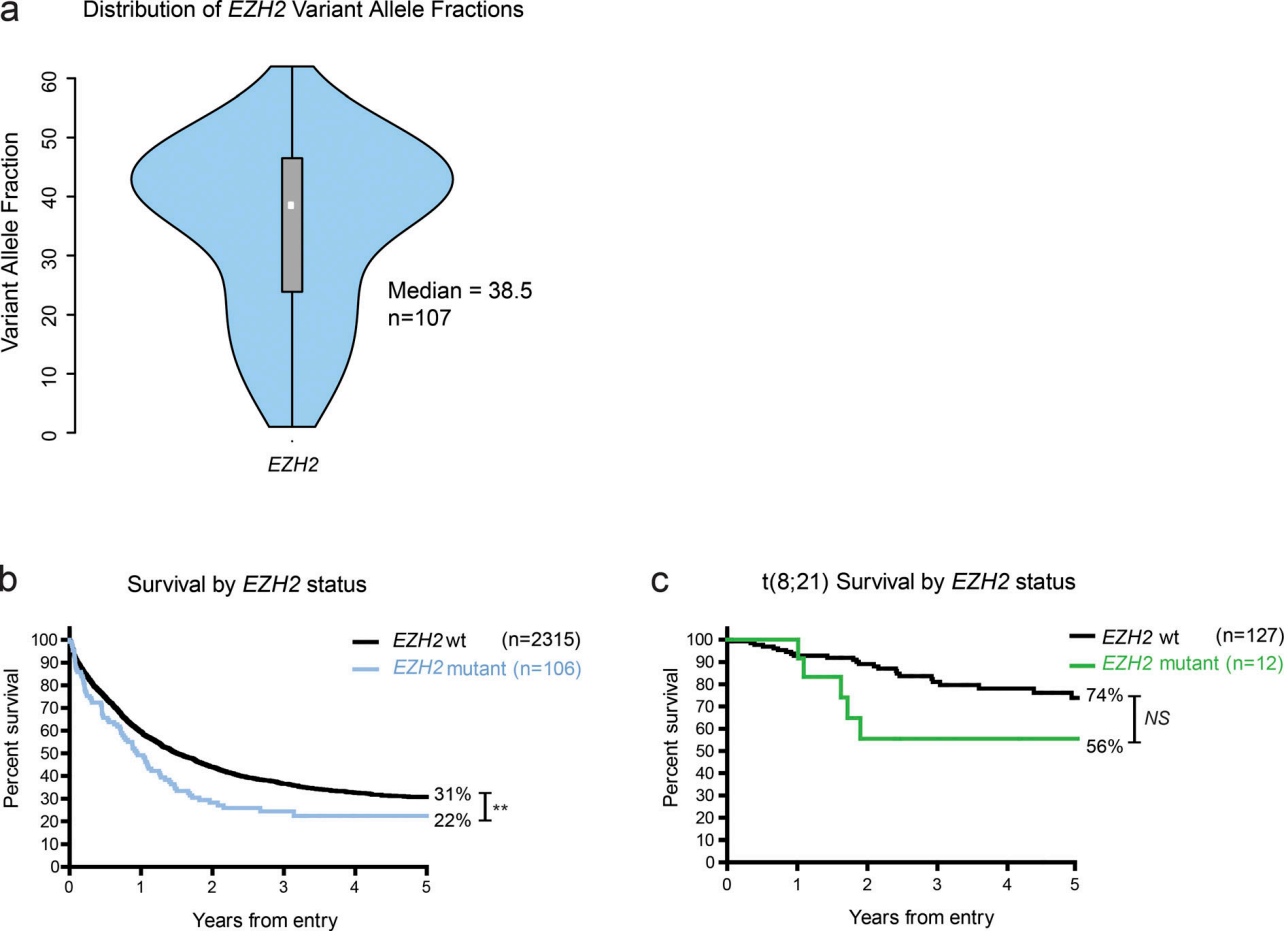
***EZH2* mutations may occur as early events in human AML and confer poor prognostic outcomes across unselected AML and within the t(8;21)-rearranged human AML subtype.** Analysis of a large AML patient cohort (*n* = 2,434) demonstrated 120 patients to have EZH2 loss-of-function mutations. **(a)** These patients demonstrated a high variant allele frequency (median 38.5%) by violin plot in keeping with EZH2 mutation occurring early in disease evolution. **(b)** OS was available for 2,421 patients. For *EZH2*-mutated AML patients (*n* = 106), OS is significantly reduced compared with *EZH2* WT (*n* = 2,315; OS, 5-yr predicted survival rate, 31% *EZH2* WT vs. 22% *EZH2* mutant; HR, 1.5; CI, 1.15–1.96; log-rank [Mantel–Cox] test P = 0.0028). **(c)** OS for *EZH2*-mutated human t(8;21) AML patients (*n* = 12) is reduced compared with *EZH2* WT (*n* = 127; OS, 5-yr predicted survival rate, 74% *EZH2* WT vs. 56% *EZH2* mutant; HR, 3.94; CI, 1.15-1.96; log-rank [Mantel–Cox] test P = 0.0503). **, P < 0.01.

We next interrogated the role of EZH2 in the preleukemic state and leukemia induction. Immunophenotyping demonstrated no significant differences in the frequency of HSC, HSPC, and lineage-negative (Lin^−^) populations within the c-kit^+^ compartment (used for the retroviral transduction assays) between normal *Ezh2^+/+^* and *Ezh2^−/−^* genotypes. Moreover, serial replating if anything was less robust in *Ezh2^−/−^* HSPC (Fig. S4 a). In vitro serial replating assays showed no difference in the clonogenic output or serial replating potential between *Ezh2^−/−^* and *Ezh2^+/+^* HSPCs transformed with *MLL-AF9* or *AML1-ETO9a*, in contrast to our maintenance experiments, where we excised *Ezh2* in an already established malignancy (Fig. S4, b and c). We next generated primary *MLL-AF9* or *AML1-ETO9a* leukemias in recipient mice using transformation of *Ezh2^−/−^* HSPCs and compared survival and phenotype to *Ezh2^+/+^* leukemias generated by the same oncogenes (Fig. 3 a). Remarkably, and diametrically opposite to its role in disease maintenance, we observed that *Ezh2* loss significantly accelerated the development of phenotypically identical *MLL-AF9* and *AML1-ETO9a* AML (Fig. 3, b–d; and Fig. S4, d–i). These data demonstrate that *Ezh2* functions as a tumor suppressor during AML induction and that its role is highly context specific during AML evolution. To further explore the role of *EZH2* mutations in human disease, we extended our analysis into a large cohort of AML patient samples (*n* = 2,434) from the National Cancer Research Institute (NCRI) AML studies (AML14, AML15, AML16, AML17, and LI1). Mutations in *EZH2,* all of which were predicted loss-of-function mutations, were detected in 107 patients (∼5%), a finding in keeping with previous series (Ley et al., 2013; Papaemmanuil et al., 2016; Saygin et al., 2018). Using the variant allele frequency to infer clonal structure, in the majority of patients, the mutations occurred within the dominant clone, with a median variant allele frequency of 0.385 (Fig. 4 a). As *EZH2* mutations are predominantly hemizygous, these data infer that the mutations occur as early events within the evolution of multiple leukemias, an observation that strongly corroborates our experimental data. We further assessed the prognostic significance of *EZH2* mutations, correlating the presence of a mutation with disease outcomes. Across the entire cohort, we could demonstrate that mutation of *EZH2* was associated with a statistically significant decrease in overall survival (OS; 5-yr predicted 31% *EZH2* WT vs. 22% *EZH2* mutant; hazard ratio [HR], 1.5; confidence interval [CI], 1.15–1.96; P = 0.0028; Fig. 4 b). However, EZH2 mutations have been previously documented to occur in older patients and those with secondary AML or “MDS-like” AML, subgroups associated with a poorer survival and less likely to receive intensive therapy (Ernst et al., 2010; Nikoloski et al., 2010; Lindsley et al., 2015; Saygin et al., 2018). To offset these potentially confounding variables, we assessed the impact of mutation in a single AML genotype with significant co-occurrence with *EZH2*, the chromosomal translocation t(8;21) subgroup that rearranges the *AML1* and *ETO* genes. Not only is this subgroup immediately relevant to our experimental data, it is also predominantly associated with younger age at presentation and a highly favorable outcome (Grimwade et al., 1998, 2010; Byrd et al., 2002; Schlenk et al., 2004; Marcucci et al., 2005). However, as the numbers of patients with co-occurrence of the *AML1-ETO* rearrangement and *EZH2* mutation in our study were small (*n* = 7), we combined our series with another series of 38 patients with t(8;21), 5 of whom also had co-occurrence of *EZH2* mutations, available from a published dataset (Faber et al., 2016). Although still limited by small numbers, our observation across all samples was replicated in this highly specific group, with *EZH2*-mutated patients demonstrating a similarly unfavorable prognosis (5-yr predicted survival rate, 74 vs. 56%), with an adverse HR for OS (HR, 3.94; CI, 1.00–1.55) and a strong trend for a lower survival advantage by log-rank analysis (P = 0.0503; Fig. 4 c). Taken all together, these data, obtained in large numbers of primary patient samples, corroborate our experimental findings on the tumor-suppressive role of EZH2 during AML induction and further implicate mutation of *EZH2* as a poor prognostic factor in AML.

### Loss of *Ezh2* derepresses specific transcriptional programs during AML evolution through loss of bivalent promoter status

**Figure 5.**
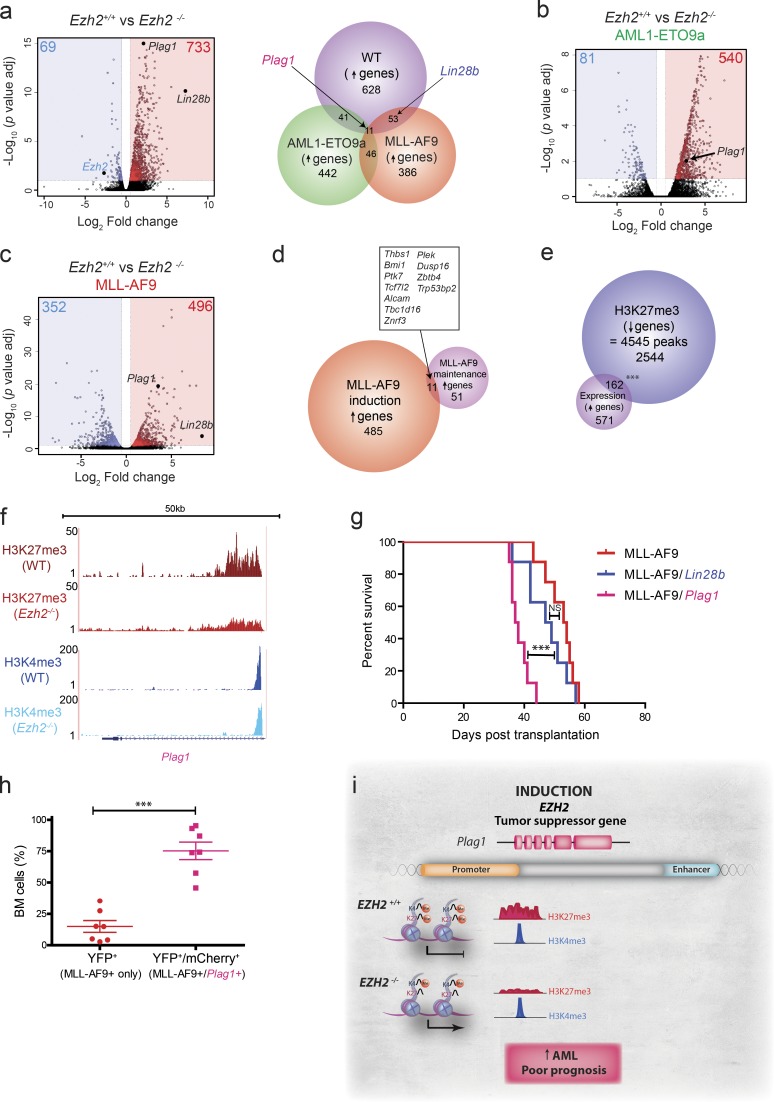
**Integrated genomic analysis provides mechanistic insights into *Ezh2*’s tumor-suppressive function in AML induction at both chromatin and molecular levels and identifies pleiomorphic adenoma gene 1 (*Plag1*) as a potent downstream mechanistic effector of *Ezh2* loss. (a)** Left: Volcano plot for *Ezh2^+/+^* vs. *Ezh2^−/−^* normal murine c-kit^+^ BM HSPCs demonstrating gene expression changes and showing the adjusted significance P value (log_10_) vs. fold change (log_2_; *n* = 2, biological replicates for *Ezh2^+/+^* and *Ezh2^−/−^*). Right: Venn diagram showing overlap of up-regulated genes following *Ezh2* loss in normal murine c-kit^+^ BM HSPCs (*n* = 733) compared with genes differentially up-regulated in *Ezh2^−/−^* MLL-AF9 murine leukemias (*n* = 496) and genes differentially up-regulated in *Ezh2^−/−^* AML1-ETO9a murine leukemias (*n* = 540). **(b)** Volcano plot for *Ezh2^+/+^* vs. *Ezh2^−/−^ AML1-ETO9a* murine leukemias demonstrating gene expression changes and showing the adjusted significance P value (log_10_) vs. fold change (log_2_; *n* = 2, biological replicates for *Ezh2^+/+^* and *Ezh2^−/−^*). **(c)** Volcano plot for *Ezh2^+/+^* vs. *Ezh2^−/−^ MLL-AF9* murine leukemias demonstrating gene expression changes and showing the adjusted significance P value (log_10_) vs. fold change (log_2_; *n* = 2, biological replicates for *Ezh2^+/+^* and *Ezh2^−/−^*). **(d)** Venn diagram of genes up-regulated following *Ezh2* loss during MLL-AF9 induction (*n* = 496) with genes up-regulated following Ezh2 inhibition in MLL-AF9 maintenance (*n* = 62) demonstrates only minimal overlap. **(e)** Overlap between down-regulated H3K27me3 peaks (*n* = 4,545, relating to 2,544 genes) and up-regulated genes (*n* = 733) following *Ezh2* loss in normal murine c-kit^+^ BM HSPCs demonstrates ∼20% of genes up-regulated are due to loss of the H3K27me3 repressive mark at chromatin (hypergeometric test P = 1.187 × 10^−18^). **(f)** ChIP-seq analysis of H3K27me3 and H3K4me3 chromatin marks reveals resolution of conflicting histone marks H3K27me3 and H3K4me3 at the bivalent promoter region in favor of gene activation for the oncogene *Plag1* in WT murine Lin^−^ BM HSPCs following *Ezh2* loss, explaining its increased expression. **(g)** Kaplan–Meier graph demonstrating significantly decreased survival for *MLL-AF9/Plag1*–overexpressing primary leukemias (*n* = 8 animals, log-rank [Mantel–Cox] test P < 0.0001) compared with MLL-AF9 primary leukemias (*n* = 8 animals, control). *MLL-AF9/Lin28b*–overexpressing primary leukemias trend toward accelerated leukemia induction (although NS). **(h)** Expansion of dual YFP (*MLL-AF9*)– and mCherry (*Plag1*)–expressing BM cells compared with single YFP (*MLL-*AF9)–expressing BM cells of terminal *MLL-AF9/Plag1* leukemias as assessed by flow cytometry (*n* = 7 leukemias in each arm shown; P < 0.0001, two-tailed *t* test). **(i)** Model (see text also). During normal hematopoiesis (*EZH2*^+/+^), EZH2 prevents the aberrant expression of oncogenes such as *Plag1* (shown) and *Lin28b* through maintenance of H3K27me3 and a bivalent state at their gene promoters (top). However, following EZH2 loss of function (*EZH2*^−/−^), loss of methyltransferase activity leads to a decrease in the H3K27me3 modification at the promoters of oncogenes such as *Plag1* and *Lin28b* and resolution of the bivalent state toward gene activation, accelerating AML induction and conferring a poor prognosis. ***, P < 0.001. All error bars are ± SEM.

To identify the oncogenic transcriptional pathways repressed by Ezh2 and the mechanisms that facilitate accelerated transformation following its loss, we performed differential global gene expression analysis using RNA-seq in *Ezh2^+/+^* and *Ezh2^−/−^* nontransformed (“premalignant”) Lin^−^ HSPCs (Fig. 5 a), comparing this to similar datasets generated in *Ezh2^+/+^* and *Ezh2^−/−^ AML1-ETO9a* and *MLL-AF9* leukemias (Fig. 5, b and c). Gene expression changes were modest and, as anticipated, *Ezh2* loss led to a greater number of genes up-regulated (derepressed) rather than down-regulated across all three conditions (premalignant, 733 up/69 down; *AML1-ETO9a*, 540 up/81 down; and *MLL-AF9*, 496 up/352 down; Tables S3, S4, and S5). Comparing the de-repressed candidate genes in each condition, within the overlapping genes (Fig. 5 a), a few select candidate genes were up-regulated across multiple conditions, notably *Plag1* (common to all), a transcription factor with known oncogenic roles in *CBFB-MYH11* murine leukemias (Castilla et al., 2004; Landrette et al., 2005), and the well-characterized oncogenic RNA-binding protein *Lin28b* (common to premalignant and *MLL-AF9* comparisons), which has also been demonstrated to play a role in the acceleration of *JAK2-V617F*–driven myelofibrosis following *Ezh2* loss (Shimizu et al., 2016). To further explain the contradictory and opposite effects of *Ezh2* loss during AML induction and maintenance, we compared genes de-repressed in established MLL-AF9 leukemias following pharmacological inhibition of Ezh2 (62 genes) to genes derepressed during the induction of *Ezh2^−/−^* MLL-AF9 leukemias (Fig. 5 d). Of note, we could only demonstrate a minimal overlap between these gene sets (11/496 genes, ∼2%) and similarly, when we compared our genes de-repressed during induction with a published dataset documenting genes de-repressed following genetic loss of *Ezh2* during the maintenance of MLL-AF9 AML, the overlap was also low (Tanaka et al., 2012; 60/496 genes, ∼12%). Thus, derepression of different gene programs explains the contrasting phenotypic outcomes of *Ezh2* loss of function in AML induction and maintenance and, together with the minimal toxicity of GSK343 in normal CD34^+^ cells (Fig. S3 d), provides some reassurances that targeting EZH2 in established AML will be a safe strategy.

Comparing chromatin immunoprecipitation sequencing (ChIP-seq) analysis in premalignant HSPC from *Ezh2^+/+^* and *Ezh2^−/−^* mice, a large number of regions demonstrated differential H3K27me3 modification (4,545 regions with decreased H3K27me3 modification and 5 regions increased). These were linked to 2,544 genes and occurred predominantly at intergenic (47%) regions and intragenic (53%) regions in keeping with enhancer elements and promoters, with 28% of the intragenic differential regions occurring at transcriptional start sites. Comparing regions with loss of H3K27me3 and up-regulation of gene expression, ∼22% of genes (162/733 including *Plag1* and *Lin28b*) overlapped, suggesting that loss of *Ezh2-*mediated methyltransferase activity was directly responsible for their up-regulation (Fig. 5 e). To further interrogate whether *Ezh2* loss impacted mainly promoter or enhancer function, we performed global differential ChIP-seq in *Ezh2^+/+^* and *Ezh2^−/−^* HSPCs for the typically enhancer-associated histone modification H3K27ac and promoter-associated H3K4me3 histone marks (Tables S6, S7, and S8). Of note, we found only 27 regions with an increase in H3K27ac following *Ezh2* loss (compared with 897 with decreased H3K27ac modification). However, in the 162 genes where up-regulated gene expression correlated with H3K27me3 loss, over half (88/162, 55%) demonstrated H3K27me loss at their promoter region. The majority of these genes (132/162, 81%; and 75/88, 85%, respectively) demonstrated bivalent promoters, with their promoters also marked by H3K4me3 in *Ezh2^+/+^* WT HSPCs. Moreover, in a small subset of genes (6/88, 7%) that included *Plag1* and *Lin28b* (Figs. 5 f and S5 a), the H3K4me3 modification signal was actually seen to significantly increase following *Ezh2* loss. These data suggest that during AML induction *Ezh2* loss does not alter enhancer function but results in the resolution of a small number of bivalent promoters, leading to derepression of their genes and up-regulation of an oncogenic program.

### Plag1 is a specific downstream mediator of *Ezh2* loss

Finally, to functionally validate candidate genes from these derepressed programs as potential mechanistic effectors of the accelerated AML induction following *Ezh2* loss in vivo, we generated retroviral constructs for *Plag1* and *Lin28b*. These genes were overexpressed along with *MLL-AF9* in WT *Ezh2^+/+^* HSPCs. Bulk-transduced populations were transplanted into recipient mice to generate primary murine leukemias (Fig. S5, b and c) and determine if coexpression of *Plag1* or *Lin28b* would phenocopy *Ezh2* loss and accelerate AML onset in comparison with *MLL-AF9* alone. Overexpression of *Lin28b* in transgenic mice results in peripheral T cell lymphomas with a relatively lengthy latency (Beachy et al., 2012), while *Plag1* overexpression alone does not induce disease (Landrette et al., 2005), so neither alone were predicted to cause AML. The *Lin28b^+^MLL-AF9* transplanted cohort demonstrated no significant difference in survival (Fig. 5 g; P = 0.22). In contrast, *Plag1* overexpression with *MLL-AF9* profoundly accelerated the development of leukemia compared with the *MLL-AF9* control arm (Fig. 5 g; P < 0.0001). Strikingly, even within the *Plag1/MLL-AF9* transplanted mice, we observed a marked expansion of the dual *Plag1/MLL-AF9* (YFP^+^/mCherry^+^)–transduced cells over the single *MLL-AF9* (YFP^+^ only)–expressing leukemia cells (from a 1:1 ratio at the time of transplant to an average of 12:1 at the time of necropsy; Fig. S5 d) when BM tissues were analyzed (Fig. 5 h), demonstrating a strong selective advantage for the combination of *MLL-AF9* and *Plag1*. These data identify *Plag1* as an oncogene whose de-repression contributes to *EZH2*-mutated AML. Moreover, neither expression of *Plag1* nor *Lin28b* was significantly altered following GSK343 treatment of MLL-AF9 tumors, reinforcing the concept of disparity between transcriptional programs repressed by *Ezh2* that are responsible for early tumor suppression and later maintenance of these leukemias.

## Discussion

The methyltransferase Ezh2 has pleiotropic effects during development and homeostasis and in pathological conditions such as cancer (Cao et al., 2002; Kuzmichev et al., 2002; Varambally et al., 2002; Kleer et al., 2003; Francis et al., 2004; Kamminga et al., 2006; Barski et al., 2007; Wagener et al., 2008; Ezhkova et al., 2009; Simon and Kingston, 2013). Our findings uncover novel and dramatically opposing functions of Ezh2 during AML evolution that are dependent upon the phase of disease, with *Ezh2* functioning as a tumor suppressor in AML induction and as a facilitator of disease in established AML. These apparently contradictory phenotypes can be explained by derepression of almost mutually exclusive transcriptional pathways normally repressed by Ezh2-mediated H3K27 methylation in each phase. These data highlight the impact of cellular context on the function of Ezh2, the PRC2 complex, and epigenetic regulators in general. To our knowledge, this is the first description of an epigenetic regulator having both tumor-suppressive and oncogenic function in different phases of the same cancer. In addition, our work further validates EZH2 as a therapeutic target in MLL-rearranged leukemias and, from the use of another mouse model and a number of different AML patient samples, extends this therapeutic potential more generally across other AML genotypes. However, loss of EZH2 protein has also been demonstrated to up-regulate HOX gene expression and to mediate resistance to certain targeted and chemotherapy agents (Göllner et al., 2017). Critically, our detailed analysis demonstrates that Ezh2 functions only as a tumor suppressor during leukemia induction and that its catalytic inhibition does not up-regulate HOX genes during AML maintenance (Table S2). These data, along with the observation that in MDS, *Ezh2* loss facilitates disease occurrence but retards progression to AML (Sashida et al., 2014), provide some reassurances that targeting EZH2 is a safe strategy in established disease.

Our findings also suggest that *EZH2* mutations confer a poor prognosis on AML patients, with this clinical observation corroborating our experimental findings during disease induction. Further supporting our findings, a similar poor prognosis has previously been described for patients with *EZH2* mutations and the related myeloid malignancies MPN and MDS (Guglielmelli et al., 2011; Bejar et al., 2012), and recent efforts to refine prognostic groups within AML (Papaemmanuil et al., 2016) have identified the chromatin and spliceosome group, in which *EZH2* mutations are grouped, as one of the groups with the poorest survival. However, as we have alluded to, *EZH2* mutations cosegregate with other poor-risk characteristics, such as patient age and an increased likelihood of antecedent myeloid malignancy such as MPN or MDS. Our studies within a single good-risk genotype, the t(8;21) subgroup, suggest that EZH2 mutation is an independent poor-risk characteristic in AML; however, further studies are necessary to determine its exact role as a prognostic factor.

The PRC2 complex, with Ezh2 as its catalytic component, is known to repress gene expression through H3K27me3 mediated effects on both proximal promoter and distal enhancer cis-regulatory elements. Although we have demonstrated that loss of H3K27me3 following loss of *Ezh2* occurs at both, the majority of regions with H3K27me3 loss occur at distal and intragenic enhancer regions. Of note, however, our data suggest that loss of Ezh2 mediates its tumor-suppressive effects predominantly through alterations of H3K27me3 at promoters rather than enhancers. During disease induction, over half of the derepressed genes lose H3K27me3 at their promoters. In contrast, only 30 derepressed genes lost H3K27me3 at enhancer elements. Moreover, global analysis of the reciprocal activation mark, H3K27Ac, demonstrated only minimal changes, and no up-regulated gene demonstrated coordinated loss of H3K27me3 and gain of H3K27Ac at an enhancer element. These findings in AML are in contrast to the changes described in MPN models, where *Ezh2* loss accelerated *Jak2*V617F-driven myelofibrosis (Shimizu et al., 2016; Yang et al., 2016). These highlight an “epigenetic switch,” where loss of H3K27me3 is accompanied by an increase in H3K27Ac following *Ezh2* loss and leukemia-initiating cells from the *Ezh2^−/−^*/Jak2V617F mice showed sensitivity to BET inhibitors, targeting gene expression related to altered H3K27Ac (Sashida et al., 2016). However, our data demonstrate that in AML, Ezh2 maintains H3K27me3 at bivalent promoters to repress specific oncogenes during disease induction. Our data are in keeping with the effects of normal and mutant Ezh2 during lymphoid development and lymphomagenesis (Béguelin et al., 2013). During normal B cell development, Ezh2 is an absolute requirement for the germinal center reaction, where it transiently halts terminal B cell development by maintaining the repressive H3K27me3 mark at a subset of bivalent promoters for genes involved in terminal B cell differentiation, such as *IRF4* and *PRDM1*. In mice overexpressing the activating mutation *Ezh2*^Y641F^, the increased catalytic activity of this mutation leads to increased H3K27me3 deposition and permanent silencing at these genes, facilitating lymphomagenesis by preventing further differentiation and perpetuating the germinal center reaction (Béguelin et al., 2013). Similarly, in the differentiation of invariant natural killer T cells (iNKTs), the balance between Ezh2-mediated methylation and Utx/Kdm6a–mediated demethylation of H3K27 at the bivalent promoter of the iNKT master regulator Plzf determines the expression of *Plzf* and the emergence of iNKTs (Beyaz et al., 2017).

Thus, our data reveal that the tumor-suppressive functions of Ezh2 are to maintain repression of a small number of oncogenes, including *Plag1* and potentially *Lin28b*, which, upon derepression, contribute to more rapid AML development (Fig. 5 i). Lin28b is an RNA-binding protein and master regulator of microRNA function, including the *Let7* family of microRNAs (Viswanathan et al., 2008; Piskounova et al., 2011). Lin28b-mediated repression of *Let7* leads to the subsequent overexpression of a number of oncogenic targets, including *Hmga2*, *Igf2bp1*, *Ras*, and *Myc*, dependent upon the cellular context (Balzeau et al., 2017). The *Lin28b*–*let7*–*Hmga2* axis has also been demonstrated to regulate fetal HSCs, and *Lin28b* was proposed as a master regulator of developmentally timed changes in HSCs and to account for their additional proliferative and self-renewal advantage over their adult counterparts (Copley et al., 2013). *Plag1,* the pleiomorphic adenoma gene 1, encodes a zinc-finger transcription factor, first described as a gene fusion partner in salivary gland pleiomorphic adenomas (Kas et al., 1997). *Plag1* and its homologue, *Plagl2*, have also previously been implicated in leukemia, being observed as candidate cooperating oncogenes in a retroviral insertion screen with the CBFβ-MYH11 product of the inv16 gene rearrangement (Castilla et al., 2004). Retroviral overexpression individually with CBFβ-MYH11 confirmed each of them to be a cooperating oncogene, and this study further demonstrated their overexpression in ∼20% of human AML samples (Landrette et al., 2005). Of note, similarly to the Lin28b–let7 axis, Plag1 also activates the IGF2 mitogenic signaling pathway (Van Dyck et al., 2007). Additionally, by linking these candidates, we could also demonstrate an up-regulation of *Plag1* expression in *Lin28b*-overexpressing HSPCs (Fig. S5 b), and recurrent rearrangements of the downstream target of the *Lin28b*–*let7* axis, *HMGA2*, also occur in salivary gland adenomas, further suggesting a degree of redundancy in the pathways activated by Lin28b and Plag1. Of note, *Lin28b*, but not *Plag1*, was also demonstrated to be up-regulated following loss of *Ezh2* during the accelerated development of *Jak2*V617F-mediated myelofibrosis (Sashida et al., 2016; Shimizu et al., 2016). Taken altogether, our study confirms that *Lin28b* appears to be a common oncogenic target across multiple myeloid malignancies and *Plag1* a specific target in AML up-regulated following *Ezh2* loss. These data also suggest that common pathway activation, such as IGF2 signaling, at least in part, underpins the accelerated leukemogenesis observed following their derepression (Fig. S5 e).

In summary our data demonstrate the context-dependent roles of epigenetic regulators during tumor evolution. Specifically, for loss of *Ezh2*, we identify chromatin-based mechanisms and candidate genes whose derepression accelerates AML generation, and we demonstrate that its mutation confers a poor prognosis on AML patients. In contrast, during AML maintenance, we functionally demonstrate that WT *Ezh2* plays an oncogenic role and show that it can be effectively therapeutically targeted to up-regulate an entirely different and tumor-suppressive program. This compartmentalized function suggests that it can be safely targeted across multiple AML genotypes.

## Materials and methods

### Mice

C57/BL6 strain mice engineered from the Wellcome Trust Sanger Institute pipeline (Skarnes et al., 2011) to have a *loxp* site flanking exon 9 of the *Ezh2* sequence in a homozygous fashion (*Ezh2^fl/fl^*) were bred in-house. Successive breedings between these and mice heterozygous for *Mx1-Cre* recombinase generated litters of *Ezh2^fl/fl^*; *Mx1-Cre* WT (*Ezh2^fl/fl^*; WT) or *Ezh2^fl/fl^*; *Mx1-Cre* heterozygous (*Ezh2^fl/fl^*; Cre^+^) mice. To induce Cre-mediated recombination, 6-wk-old *Ezh2^fl/fl^*; Cre^+^ and *Ezh2^fl/fl^*; WT mice were administered five doses of pIpC (300 µg per dose; Sigma) via intraperitoneal injection on alternate days over 10 d. All experiments were performed using littermates unselected for gender but age matched from date of birth and timing of pIpC administration. Peripheral blood was collected and counted as previously described (Chan et al., 2011). Excision efficiency was characterized by quantitative PCR (qPCR) of genomic DNA extracted from whole blood or BM. All mice were housed in a specific pathogen–free animal facility allowing unrestricted access to food and water, and all experiments were conducted under UK Home Office regulations under a UK Home Office project license. This research has been regulated under the Animals (Scientific Procedures) Act 1986 Amendment Regulations 2012 following ethical review by the University of Cambridge Animal Welfare and Ethical Review Body.

### Isolation of murine cell populations

For the transplantation experiments, mice were euthanized humanely and both femurs and tibias harvested. These were flushed with sterile PBS, yielding BM cells that were then filtered through a 70-µm EASYstrainer (Greiner). RBCs were lysed using RBC lysis buffer (5 PRIME). For the serial replating and transplantation experiments, BM cells from *Ezh2^fl/fl^*; WT or *Ezh2^fl/fl^*; Cre^+^ were selected for cell-surface c-kit expression using CD117 MicroBeads (Miltenyi Biotec) according to the manufacturer’s protocol. For the *Lin28b/Plag1* in vivo validation experiments, c-kit–positive BM cells from 8–12-wk-old C57/BL6 WT mice were isolated in the same way.

For the ChIP-seq experiments, Lin^−^ HSPCs from whole murine BM were isolated using a Lineage Cell depletion kit (Miltenyi Biotec) as per the manufacturer’s protocol.

### Mouse transplantation experiments

For the induction experiments, retrovirally transduced *Ezh2^fl/fl^*; WT or *Ezh2^fl/fl^*; Cre^+^ BM cells (both pretreated with pIpC to induce *Ezh2* deletion in the Cre^+^ arm) were generated, yielding *Ezh2^+/+^* or *Ezh2^−/−^* states respectively. After assessment of transduction efficiency, 0.2–1.5 × 10^6^ bulk BM cells from each arm (containing an equal number of positively transduced cells) were transplanted into age-matched lethally irradiated (two doses of 5.5 Gy each) C57/BL6 WT recipient mice via tail vein injection, generating *Ezh2*^+/+^ and *Ezh2*^−/−^ leukemia arms for each oncogene. Similarly, for the maintenance experiments, to generate primary leukemias, retrovirally transduced *Ezh2^fl/fl^*; Cre^+^ BM cells (obtained from 8–12-wk-old mice) were transplanted via tail vein injection into lethally irradiated (two doses of 5.5 Gy each) C57/BL6 WT recipient mice. For secondary leukemias, 10^6^ bulk splenocytes harvested from primary leukemic mice were transplanted into age-matched lethally irradiated C57/BL6 WT recipient mice via tail vein injection, and pIpC or an equal volume of PBS (control) was administered intraperitoneally following engraftment.

### Histopathology

All tissues were fixed, embedded, and section as described previously (Giotopoulos et al., 2015).

### Western blotting

Western blotting was performed using 12% SDS-PAGE gels and standard protocols. The antibodies used were anti-Tri-Methyl-Histone H3 (9733; Cell Signaling Technology) and anti-Tubulin (44928; Abcam). Secondary antibodies were IRDye 680RD and IRDye 800CW (LI-COR Biosciences). An Odyssey Infrared Imaging system (LI-COR Biosciences) was used to scan the immunoblots.

### Serial replating assays

Normal or immortalized mouse BM cells were plated at concentrations of 3.3–5 × 10^4^/plate (in duplicate) using MethoCult GF M3434 (STEMCELL Technologies) methylcellulose medium to assess myeloid potential. Colonies were scored manually (and/or using an automated reader, STEMvision; STEMCELL Technologies), total cell numbers were measured at 7 d, and equal numbers of cells were replated using the same conditions.

### Retroviral transduction assays

For virus production, TransIT-LT1 transfection reagent (Mirus) was used to transfect the individual retroviral vectors MSCV-MLL-AF9-IRES-YFP, MSCV-AML1-ETO9a-IRES-GFP, MSCV-Lin28b-IRES-mCherry, MSCV-Plag1-IRES-mCherry, p-babe-puro, or p-babe-Cre-puro and the psiEco packaging plasmid in a 1:1 ratio into 293T cells according to the manufacturer’s protocol. Supernatant was harvested 48 and 72 h after transfection. One million cells were resuspended into 1 ml retroviral supernatant supplemented with mIL-3, IL-6, and murine stem cell factor cytokines (final concentrations of 10 ng/ml, 10 ng/ml, and 100 ng/ml respectively; Peprotech) and polybrene to a final concentration of 8 ng/µl. Cells were spinoculated with retroviruses as previously described (Giotopoulos et al., 2016).

### EPZ6438 in vivo dosing experiments

AML1-ETO9a AML murine leukemias were injected intravenously into lethally irradiated C57BL/6 (CD45.1) congenic recipients (2 million AML cells and 300,000 CD45.1 BM helper cells/mouse). Upon disease dissemination, EPZ-6438 (Insight Biotechnology) or vehicle (1% DMSO, 0.5% carboxymethylcellulose sodium salt, and 0.1% Tween-80 in water) was administered daily by oral gavage (160 mg/kg).

### Cell culture and inhibitor assays

Kasumi-1, KG1, K562, MOLM-13, and MV411 cell lines were grown in RPMI-1640 medium supplemented with FBS and penicillin/streptomycin (10–20% and 1% final concentration, respectively; Sigma-Aldrich). Splenocytes from leukemic MLL-AF9 mice were passaged in X-Vivo 20 culture medium (Lonza) supplemented with mIL-3, IL-6, and murine stem cell factor cytokines (final concentrations of 10 ng/ml, 10 ng/ml, and 50 ng/ml respectively; Peprotech). Human AML cell lines, mouse tumors, and human primary AML cells were plated in duplicate in methylcellulose medium. For AML cell lines, 30,000 cells per plate were used. For murine MLL-AF9 tumors, 50,000 splenocytes per plate were used. For primary AML samples, 50–80,000 cells per plate were used. For AML cell lines and primary AML samples, MethoCult H4435 (STEMCELL Technologies) was used, and for murine cells, MethoCult GF M3434 was used in the presence of DMSO or the small-molecule EZH2 inhibitor GSK343 (10 µM). Colonies were scored at 7–12 d.

### Cell proliferation and liquid culture assays

Kasumi-1 cells cultured in the conditions detailed above were plated at concentrations of 0.5 × 10^6^/ml into 12-well plates, to which DMSO or GSK343 (10 µM) was added, mixed, and incubated. Cells were counted using a CASY Counter (Schärfe System GmbH) at 72 h, resuspended in fresh media, and returned to the original concentration. This was repeated at 144, 216, and 288 h, and total cell numbers were counted. The CellTiter 96 AQueous One Solution Cell Proliferation Assay (Promega) was used to assay the antiproliferative and cytotoxic effects of GSK343 against human AML cell lines and mouse leukemic cells as per the manufacturer’s protocol. Proliferation curves and IC_50_ values were generated using Prism statistical software.

### Human primary AML sample collection, processing, and sequencing

Peripheral blood/BM samples were collected from newly diagnosed/relapsed patients with AML into cytogenetic media (RPMI supplemented with Hepes, lithium heparin, and gentamicin). This was part of the Causes of Clonal Blood Cell Disorders study (Department of Haematology, University of Cambridge) approved by the Cambridge and Eastern Region Ethics Committee. Patients gave written informed consent, and research was performed in accordance with the Declaration of Helsinki. All samples were anonymized and identifiable only by a tissue bank code. The mononuclear fraction was obtained following a 1:1 dilution in PBS and layering onto 1:1 volume of Lymphoprep (Axis-Shield). Samples were centrifuged for 45 min at 1,400 rpm, and the mononuclear fraction was carefully removed using a 1-ml pipette. After washing in MACS buffer (500 ml PBS without Ca/Mg, 2.5 g BSA, and 2 ml of 0.5 M EDTA, filter sterilized), cells were counted for use. For assessment of EZH2 mutation status, DNA from AML samples from the UK NCRI AML study group trials AML 14 (ISRCTN62207270/ClinicalTrials.gov number NCT00005823), AML15 (ISRCTN17161961/EudraCT number 2005–001149-40), AML16 (ISRCTN 11036523/ClinicalTrials.gov number NCT00454480), AML17 (ISRCTN31682779/EudraCT number 2013–002730-21), AML18 and LI1 (ISRCTN40571019) were sequenced using solution-based capture hybridization and next generation sequencing using a captured based panel of 126 genes. Variants in EZH2 were called using established in house algorithms and manual variant annotation was conducted to retain putative oncogenic variants as previously described (Papaemmanuil et al., 2016). The average depth over the EZH2 locus was 230× and for the reported mutations variant allele fractions were corrected for local copy-number changes as well as focal regions of homozygosity. Clinical outcome data were available for 2,421 cases in our study. In addition to the case studies as part of the UK NCRI trials, mutation and outcome data were available from a further published dataset of 38 AML1-ETO patients that contained five patients with EZH2 mutations and AML1-ETO rearrangements (Faber et al., 2016).

### Flow cytometry

Single-cell suspensions of BM or spleen were prepared as previously described (Chan et al., 2011). All analyses considered only 7-AAD^−^ (BD) populations. Annexin V (APC conjugated; BD) and 7-AAD were used in cell viability assays according to the manufacturer’s protocol. For cell cycle analysis, cells were washed in PBS, fixed in 70% ethanol in PBS and resuspended in 50 µg/ml propidium iodide with 0.1 mg/ml RNase then incubated at 37°C for 30 min. To assess H3K27me3 status following Ezh2 inhibition with GSK343, 8 × 10^6^ *Ezh2^fl/fl^*; WT MLL-AF9 AML splenocytes from two tumors were passaged (in duplicate) in X-Vivo 20 media at 0.5 × 10^6^/ml concentrations. At 0 h, DMSO and GSK343 (10 µM) were added to each MLL-AF9 tumor. At 24, 48, and 72 h, 10^6^ cells from each condition were resuspended in BD Cytofix/Cytoperm solution (BD Biosciences) and incubated and washed with 10× BD Perm/Wash cell permeabilizing buffer (BD Biosciences) as per the manufacturer’s protocol. Cells were then resuspended in BD Perm/Wash buffer containing either H3K27me3–Alexa Fluor 647–conjugated antibody (1:100 dilution; Abcam) or Histone H3–Pacific Blue–conjugated antibody (1:50 dilution; Cell Signaling Technology). At each time point, remaining cells were washed and returned to 0.5 × 10^6^/ml concentration with fresh media. Unstained cells were used to gate live cells. GFP/YFP and mCherry were integrated into several DNA constructs used across retroviral transduction and overexpression experiments. Assessment of these markers by flow cytometry across MLL-AF9 (YFP) or AML1-ETO9a (GFP)–transformed murine c-kit^+^ BM cells, mononuclear cells obtained from blood samples of mice with these leukemias, and tissues isolated from terminal leukemic mice allowed for an estimation of the percentage of transformed cells or leukemic burden to be made. The mCherry marker was used in the MSCV-IRES-mCherry constructs that candidate genes *Lin28b* and *Plag1* were cloned into for functional assessments of expression. Flow cytometry was performed on a BD LSRFortessa cell analyzer, and all data were analyzed with FlowJo software (Tree Star).

### Quantitative real-time PCR (qRT-PCR)

Total RNA was isolated using an AllPrep DNA/RNA Mini kit (QIAGEN). cDNA was then prepared from 0.5 µg RNA using the SuperScript III Reverse transcription Kit (Invitrogen). qRT-PCR was performed on diluted cDNA (1:10 in water) using Brilliant III Ultra-Fast QPCR Master Mix (Agilent) and gene-specific primers (Sigma-Aldrich) on an MX3000p qPCR system (Agilent) and standard cycling setup. The following primer sequences were determined from Primer3 (http://primer3.ut.ee): Lin28b forward, 5′-ATG​GCA​CTT​CTT​TGG​CTG​AG-3′; Lin28b reverse, 5′-ATA​GGT​GGA​GAC​GGC​AGG​AT-3′; Plag1 forward, 5′-GAC​AAG​GCC​TTT​AAC​AGT​GTT​G-3′; and Plag1 reverse, 5′-TCA​GGA​GAG​TGA​GTA​GCC​ATG-3′.

### RNA-seq

Total RNA was extracted from Lin^−^ BM cells isolated from 6-wk-old *Ezh2^fl/fl^*; Cre^+^ and *Ezh2^fl/fl^*; WT mice after pIpC treatment using an AllPrep DNA/RNA Mini Kit as per the manufacturer’s protocol. RNA was also extracted from unfractionated BM of MLL-AF9 and AML1-ETO9a malignant *Ezh2^+/+^* and *Ezh2^−/−^* mice and from malignant splenocytes from *Ezh2^+/+^* MLL-AF9 mice propagated and cultured in vitro 24 h after DMSO and GSK343 treatment. RNA was quantified using a NanoDrop 1000 Spectrophotometer (Thermo Scientific). For each experiment, 5 µg RNA was used for library preparation using a Rapid Directional RNA-Seq Kit (NEXTflex). Library quality was checked, and barcoded libraries were pooled together and sequenced at the Cancer Research UK (CRUK) Cambridge Institute genomics core. Paired end RNA-seq reads were quality filtered and mapped using STAR3 against the mouse genome (mm10). Read counts were quantified with HTSeq (Anders et al., 2015) and differential expression analysis was performed with these counts using Bioconductor package DESeq2 (Love et al., 2014). Features with adjusted P value <0.05 and absolute logarithmic (base 2) fold change >0.5 were considered as having significantly altered expression.

### ChIP-seq

Chromatin immunoprecipitation was performed on murine Lin^−^ BM HSPCs as previously described (Horton et al., 2017). Cells were cross-linked with 1% formaldehyde for 5 min for histone markers (H3K27me3, H3K27Ac, and H3K4me3; Millipore, Abcam, and Diagenode, respectively). ChIP-seq library preparation of ChIP DNA or input DNA was performed using TruSeq ChIP Sample Prep Kit (Illumina). KAPA Library Quantification kit (Kapa Biosystems) was used for library DNA quantification. Average library size was determined using an Agilent DNA 1000 Kit (Agilent Technologies) run on a 2100 Bioanalyzer System (Agilent Technologies). Libraries were then pooled for multiplexing for single-read sequencing on an Illumina HiSeq 4000 machine at the Genomics Core, CRUK Cambridge Institute. Experiments were performed in duplicate on biologically independent samples.

### ChIP-seq data analysis

Adapter sequences were trimmed for all reads and mapped against the mm10 reference genome using Bowtie2 (Langmead and Salzberg, 2012). Uniquely mapped reads were retained, and peaks were called using SICER (Xu et al., 2014) with W200 and G600 parameters. Peaks in intergenic regions were assigned to genes if they were within the 100-kb window from the transcriptional start site. Differential binding analysis was performed using DiffBind (Ross-Innes et al., 2012). Overlapping analysis of peaks was performed using intersectbed from bedtools (Quinlan and Hall, 2010). Data were displayed as UCSC genome browser custom tracks. After peak calling, differentially bound histone peak lists generated from *Ezh2*^+/+^ and *Ezh2^−/−^* conditions were filtered to select all those peaks with a false discovery rate of <0.01. Features with fold changes >1.5 were considered to have significant differential binding.

### Statistical analysis and reproducibility

Unless otherwise stated, all statistical analyses used Student’s two-tailed *t* test on raw data. P values ≤0.05 were considered statistically significant. Survival curves were constructed using the Kaplan–Meier method, and statistical significance was calculated using log-rank analysis. The number of independent experiments and mice in transplantation experiments used to generate statistically significant data are detailed in the relevant figure legends.

### Data availability

All RNA-seq and ChIP-seq data have been deposited in the Gene Expression Omnibus database under the accession no. GSE112724.

### Online supplemental material

Fig. S1 shows transduction efficiencies and Cre-recombinase toxicity in *Ezh2^wt/wt^* and *Ezh2^fl/fl^* MLL-AF9–transformed cell lines (related to Fig. 1, b–d). Fig. S2 shows disease parameters in AML1-ETO9a secondary murine leukemias (related to Fig. 1 f). Fig. S3 shows GSK343 activity against human AML cell lines, immortalized murine cell lines, and primary human CD34^+^/AML samples (related to Fig. 2). Fig. S4 shows the HSC and HSPC BM composition of *Ezh2^−/−^* mice, serial replating assays, and disease parameters for MLL-AF9 and AML1-ETO9a murine leukemias for the EZH2 is a tumor suppressor during AML induction... section (related to Fig. 3). Fig. S5 provides additional information on the functional validation of *Plag1* and *Lin28b* genes as downstream mechanistic effectors of Ezh2 loss and a proposed molecular model of its downstream effects. Table S1 lists the causes of death for animals on the AML1-ETO9a maintenance experiments. Table S2 lists the differentially expressed genes in *Ezh2^+/+^* MLL-AF9 mouse tumors treated with GSK343 (vs. DMSO). Table S3 lists the differentially expressed genes in *Ezh2^+/+^* vs. *Ezh2^−/−^* Lin^−^ BM cells (HSPCs). Table S4 lists the differentially expressed genes in *Ezh2^+/+^* vs. *Ezh2^−/−^* AML1-ETO9a BM cells. Table S5 lists the differentially expressed genes in *Ezh2^+/+^* vs. *Ezh2^−/−^* MLL-AF9 BM cells. Table S6 lists the differentially bound peaks for H3K27me3 between *Ezh2^+/+^* and *Ezh2^−/−^* BM HSPCs. Table S7 lists the differentially bound peaks for H3K4me3 between *Ezh2^+/+^* and *Ezh2^−/−^* BM HSPCs. Table S8 lists the differentially bound peaks for H3K27Ac between *Ezh2^+/+^* and *Ezh2^−/−^* BM HSPCs.
